# MLVA-16 Genotyping of *Brucella abortus* and *Brucella melitensis* Isolates from Different Animal Species in Egypt: Geographical Relatedness and the Mediterranean Lineage

**DOI:** 10.3390/pathogens9060498

**Published:** 2020-06-22

**Authors:** Gamal Wareth, Mohamed El-Diasty, Falk Melzer, Gernot Schmoock, Shawky A. Moustafa, Mohamed El-Beskawy, Dali F. Khater, Mahmoud E.R. Hamdy, Hoda M. Zaki, Ana Cristina Ferreira, Loukia V. Ekateriniadou, Evridiki Boukouvala, Mostafa Y. Abdel-Glil, Ahmed M.S. Menshawy, Marta Pérez Sancho, Sonia Sakhria, Mathias W. Pletz, Heinrich Neubauer

**Affiliations:** 1 Friedrich-Loeffler-Institut, Institute of Bacterial Infections and Zoonoses, Naumburger Str. 96a, 07743 Jena, Germany; falk.melzer@fli.de (F.M.); gernot.schmoock@fli.de (G.S.); Mostafa.AbdelGlil@fli.de (M.Y.A.-G.); heinrich.neubauer@fli.de (H.N.); 2 Institute for Infectious Diseases and Infection Control, Jena University Hospital, Am Klinikum 1, 07747 Jena, Germany; mathias.pletz@med.uni-jena.de; 3 Faculty of Veterinary Medicine, Benha University, Moshtohor, Toukh 13736, Egypt; dr.shawky.gesriha@gmail.com; 4 Department of Brucellosis, Animal Health Research Institute, P.O. Box 264-Giza, Cairo 12618, Egypt; dr_mesbah_m@yahoo.com (M.E.-D.); dr.daliakhater@gmail.com (D.F.K.); hodamzaki2010@yahoo.com (H.M.Z.); 5 Department of Animal Medicine, Faculty of Veterinary Medicine, Matrouh University, Matrouh 51744, Egypt; melbeskawy@gmail.com; 6 National Reference Laboratory for Brucellosis, National Institute of Agrarian and Veterinary Research (INIAV, IP), 157 Oeiras, Portugal; cristina.ferreira@iniav.pt; 7 Faculdade de Ciências, Universidade de Lisboa, Biosystems and Integrative Sciences Institute (BioISI), Edificio TecLabs, Campus da FCUL, Campo Grande, 1749-016 Lisbon, Portugal; 8 Veterinary Research Institute, Hellenic Agricultural Organization- DEMETER, 54124 Thessaloniki, Greece; ekateriniadou@vri.gr (L.V.E.); boukouvala@vri.gr (E.B.); 9 Faculty of Veterinary Medicine, Beni-Suef University, Shamlaa Street, Beni-Suef 62511, Egypt; elmenshawy81@yahoo.com; 10 Centro VISAVET, Universidad Complutense de Madrid, Avenida Puerta de Hierro, s/n, PC 28040 Madrid, Spain; maperezs@visavet.ucm.es; 11 Departamento de Sanidad Animal, Facultad de Veterinaria, Universidad Complutense de Madrid, 28040 Madrid, Spain; 12 Institute of Veterinary Research of Tunisia, University of Tunis El Manar, Tunis 1006, Tunisia; sakhrias@yahoo.fr

**Keywords:** *B. melitensis*, *B. abortus*, MLVA-16, genotyping, Egypt, Mediterranean Basin

## Abstract

Brucellosis is a common zoonotic disease in Egypt. However, there are limited data available on the genetic diversity of brucellae circulating in Egypt and other Mediterranean areas. One hundred and nine *Brucella* (*B*.) strains were isolated from different animal species in thirteen Egyptian governorates. Multi-locus variable number tandem repeats (VNTRs) analysis (MLVA-16) was employed to determine the geographical relatedness and the genetic diversity of a panel of selected Egyptian strains (*n* = 69), with strains originating from Italy (*n* = 49), Portugal (*n* = 52), Greece (*n* = 63), and Tunisia (*n* = 4). Egyptian *B. melitensis* strains clustered into two main clusters containing 21 genotypes. Egyptian *B. abortus* strains clustered into three main clusters containing nine genotypes. The genotypes were irregularly distributed over time and space in the study area. Egyptian strains of *B. melitensis* showed MLVA-16 patterns closer to that of Italian strains. Egyptian *B. abortus* strains isolated from cattle share the same genotype with strains from Portugal and similar to strains from Italy with low genetic diversity. Strains with similar MLVA patterns isolated from different governorates highlight the movement of the pathogen among governorates. Hence, it may also reflect the long endemicity of brucellosis in Egypt with earlier dispersal of types and great local genetic diversity. Open markets may contribute to cross-species transmission and dissemination of the new types nationwide. The presence of West Mediterranean lineages of *B. melitensis* and relatedness of *B. abortus* strains from the studied countries is a result of the socio-historical connections among the Mediterranean countries. Transnational eradication of brucellosis in the Mediterranean basin is highly demanded.

## 1. Introduction

Brucellosis is a worldwide distributed zoonotic bacterial disease affecting a wide range of mammals including humans [1]. The members of the genus *Brucella* are able to circumvent the host immune system, survive, and multiply inside the phagocytic cells. Brucellosis is uncontrolled in wildlife, and the fear that it might be used in bioterrorism still exists [2]. Out of 12 phenotypically recognized species, *B. melitensis*, *B. suis*, *B. abortus*, and *B. canis* are well-known human pathogens [3,4]. *Brucella melitensis* is considered the most virulent species causing severe disease in humans. *B. suis*, except biovar 2, and *B. abortus* provoke milder illness [5]. Small ruminants and bovines are the predominant hosts for *B. melitensis* and *B. abortus*, respectively, while cross-species transmission has been proved [6,7]. *Brucella melitensis* was isolated from untypical hosts like freshwater fish [8]. *Brucella abortus* was also isolated from wildlife [9] as well as from accidental carriers hosts such as dogs and cats [10]. The epidemiological situation of brucellosis in the Middle East, in the Mediterranean basin, Africa, Asia, and in some areas of Latin America is alarming [11]. The World Organization for Animal Health (OIE), Food and Agriculture Organization (FAO), and world health organization (WHO) consider brucellosis a significant public health problem [12]. Thus, an epidemiological focus exists in the Mediterranean basin, and its eradication is challenging due to the financial restrictions in non-EU countries.

In 1939, the disease was reported for the first time in a scientific report in Egypt; however, archeological evidence has found that it has been endemic in Egypt for thousands of years and in the Mediterranean basin since at least the Middle Ages [13,14,15,16,17]. These findings confirm the continuing circulation of this zoonotic infection over centuries. Since the 1960s, when Friesian cows were imported to Egypt to increase the profit of newly established, highly intensive governmental farms, the incidence of brucellosis in cattle has increased steadily. Since then, the disease has been recognized as one of the most important livestock diseases in the country with veterinary and public health significance [17]. The unofficial movement of cattle, camels, sheep, and goats either for grazing or for trade enhances the spread of the disease across national borders and within governorates [18]. *Brucella melitensis* biovar (bv) 3 is the biovar most often isolated from humans and livestock in the Mediterranean region [12] and is responsible for most animals and human cases in Egypt followed by *B. abortus* bv1 [17,19].

Molecular typing of *Brucella* strains can be used for trace-back and trace-forward investigations as well as identification of the spreading route. Thus, the current study aimed at the investigation of the genetic diversity and the geographical distribution of the most predominant *B. melitensis* and *B. abortus* strains circulating in animals of different geographic areas of Egypt by using multiple locus of variable number tandem repeats analysis (MLVA-16) as well as to investigate their epidemiological and geographic relationship with Mediterranean strains isolated from Italy, Greece, Portugal, and Tunisia.

## 2. Results

### 2.1. Biotyping and Origin of B. melitensis and B. abortus Isolates

As shown in Table 1, a total of 109 *Brucella* isolates were determined and analyzed in this work. The isolates were identified with MALDI-TOF analysis, standard biochemical tests, and AMOS-PCR. Eighty-five strains isolated from cattle, buffaloes, sheep, goats, and camels were identified as *B. melitensis*, and 24 strains isolated from cattle, buffaloes, one dog, and one cat (kept on a dairy and cattle farm) were identified as *B. abortus*. No other *Brucella* spp. were found. The presence of both *B. melitensis* and *B. abortus* in non-preferential host species existed. The AMOS-PCR provided clear identification of strains at the species level, and the score values of all strains were higher than 2.300 in the MALDI-TOF analysis. All sampled governorates were previously reported as endemic areas of brucellosis. Most of *B. abortus* and *B. melitensis* isolates were isolated from cattle (*n* = 52) followed by buffaloes, sheep, and goats (Table 1).

### 2.2. Clustering, Diversity, and Distribution of B. melitensis

**Figure 1:**
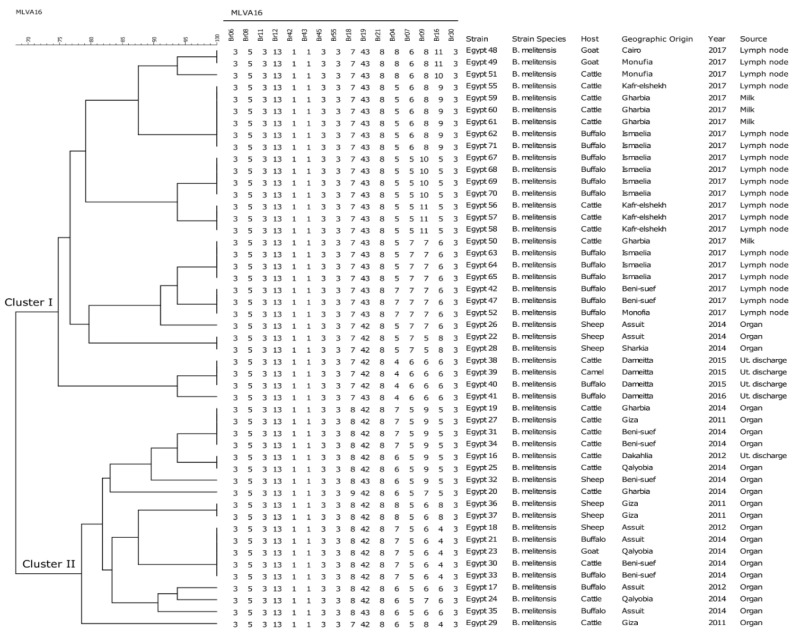
Dendrogram based on MLVA-16 genotyping UPGMA (unweighted pair group method with arithmetic mean) showing the relationship between 49 *B. melitensis* isolates recovered from different animal species in Egypt. The columns show MLVA-16 profiles of strains, identification numbers, species, host, geographic origin, year, and source of isolation.

Only 69 Egyptian strains (49 *B. melitensis* and 20 *B. abortus*) were used in the comparison. While the other 40 strains were excluded from the comparison, because the number of repeats could not be determined and were missed in at least two loci in each strain (Table S1). Thus, those will be subject to the Whole genome sequencing (WGS). Forty-nine *B. melitensis* Egyptian isolates were clustered into two main clusters containing 21 different genotypes by MLVA-16 analysis. All loci of the panel-1, locus *Bruce*21 of panel-2A, and locus *Bruc*e30 of panel-2B were homogeneous. In contrast, the most discriminatory loci were *Bruc*e18 and *Bruc*e19 (panel-2A), and *Bruc*e 04, *Bruc*e07, *Bruc*e09, and *Bruce*16 from panel-2B. The dendrogram of the genetic relatedness of *B. melitensis* strains is depicted in Figure 1. The distances between strains within the cluster and between two clusters was calculated based on the number of different and identical VNTRs. Considering a similarity cutoff value of 68% (minimum three and maximum six different loci), *B. melitensis* isolates were grouped into two major clusters. The similarity matrix of *B. melitensis* isolates is shown in Figure S1. *Brucella melitensis* strains isolated from cattle (Egypt 38), camel (Egypt 39), and buffalo (Egypt 40) at Dameitta governorate in the year 2015 showed the same genotype. A *B. melitensis* strain isolated from cattle at Gharbia governorate in 2014 (Egypt 19) had the same genotype as two strains recovered from two cattle at Beni-suef governorate in 2014 (Egypt 31, 34) and a strain recovered from cattle at Giza governorate in 2011 (Egypt 27). A *B. melitensis* isolated from cattle at Dakahlia governorate in 2012 (Egypt 16) had the same genotype as a strain of *B. melitensis* recovered from cattle in 2014 (Egypt 25) at Qalyobia governorate. *Brucella melitensis* strains isolated from sheep in 2012 (Egypt 18), and buffalo in 2014 (Egypt 21) at Assuit governorate had the same genotype as *B. melitensis* bv3 isolated from cattle and buffalo in 2014 (Egypt 30 and 33) at Beni-suef governorate as well as the same genotype of *B. melitensis* isolated from goat (Egypt 23) in 2014 at Qalyobia governorate. Several strains isolated from different hosts at different governorates in 2017 shared the same MLVA-16 profiles. For instance, two strains isolated from goats (Egypt 48, 49) in Cairo and Monufia had the same genotype. Two strains isolated from buffaloes (Egypt 42, 47) at Beni-suef shared the same genotype of a strain recovered from buffalo (Egypt 52) at Monufia. Three strains (Egypt 59, 60, 61) isolated from three cows in the same outbreak at Gharbia are similar to a strain isolated from cattle at Kafr-elshekh (Egypt 55) and two strains isolated from buffaloes (Egypt 62, 71) at Ismaelia. Three strains (Egypt 63, 64, 65) isolated from three buffaloes in the same outbreak at Ismaelia had the same genotype as a strain isolated from cattle (Egypt 50) at Gharbia. Two *B. melitensis* bv2 and bv3, isolated from sheep in 2011 (Egypt 36, 37) at Giza governorate, were found to also have the same genotype. *Brucella melitensis* bv3 strains isolated from sheep at Sharkia governorate (Egypt 28) presented the same genotype as *B. melitensis* bv2 isolated from sheep in the same year (Egypt 22) at Assuit governorate (Figure 1).

### 2.3. Clustering, Diversity, and Distribution of B. abortus

**Figure 2:**
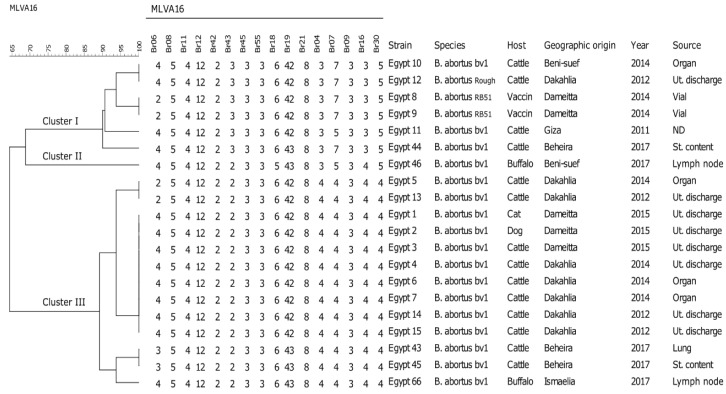
Dendrogram based on MLVA-16 genotyping (UPGMA method) showing the relationship between 20 *B. abortus* isolates recovered from different animal species in Egypt. The columns show MLVA-16 profiles of strains, identification numbers, species, host, geographic origin, year, and source of isolation.

Twenty *B. abortus* Egyptian isolates clustered into two main clusters (I and III) and one strain with a singleton genotype (cluster II), producing collectively nine different genotypes after MLVA-16 analysis. The dendrogram of the genetic relatedness of *B. abortus* strains is depicted in Figure 2. The distances between strains within the cluster and between two clusters was calculated based on the number of different and identical VNTRs. Considering a similarity cutoff value of 64% (minimum four and maximum seven different loci), *B. abortus* isolates were grouped into three major clusters. The similarity matrix between *B. abortus* isolates is shown in Figure S2. All loci from panel-1 except *Bruc*e 06 and *Bruc*e 43 were monomorphic. Also, locus *Bruce*21 from panel-2A and locus *Bruce*09 from panel-2B were monomorphic, and no difference was found at all. In contrast, *Bruc*e06 and *Bruc*e43 from panel-1, *Bruc*e18 and *Bruc*e19 from panel-2A, and *Bruc*e04, *Bruc*e07, *Bruc*e16, and *Bruce*30 from panel-2B were heterogeneous. As shown in Figure 2, a *B. abortus* rough strain isolated from cattle in 2012 (Egypt 12) at Dakahlia governorate had the same genotype of a bv1 strain recovered from cattle in 2014 (Egypt 10) at Beni-suef governorate and were similar to *B. abortus* RB51 cultured from vaccine vials with very low genetic diversity (*Bruce*06: 2→4). We observed that DNA of *B. abortus* RB51 extracted directly from vials had two repeat units less in loci *Bruce*06 than DNA extracted from cultured strains from the same vials. Two *B. abortus* bv1 isolates from cattle in 2012 (Egypt 13) and 2014 (Egypt 5) at Dakahlia governorate had the same genotype. Five *B. abortus* bv1 strains isolated from cattle in 2012 (Egypt 14, 15) and 2014 (Egypt 4, 6, 7) from the same herd at Dakahlia governorate had the same genotype as three strains isolated from a cat, a dog, and a cow in 2015 (Egypt 1, 2, 3) at Dameitta governorate (Figure 2).

### 2.4. Geographical Relatedness and the “Mediterranean Lineage”

**Figure 3:**
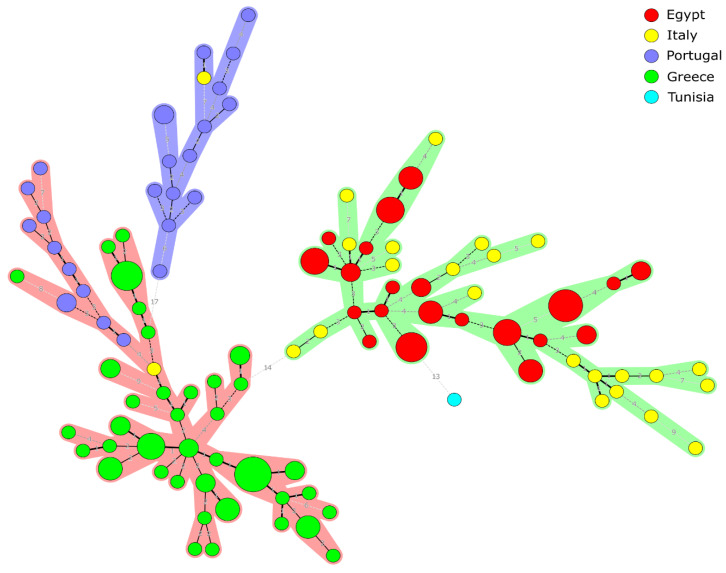
MLVA-16 minimum spanning tree describing the relationships of 163 *B. melitensis* isolates. Circles represent MLVA-16 genotypes, colored according to the country of origin, and the size of the circle indicates the number of strains with that genotype.

The MLVA-16 profiles of the 163 *B. melitensis* strains from Egypt (*n* = 49), Italy (*n* = 24), Portugal (*n* = 26), Greece (*n* = 63), and one strain from Tunisia were investigated and compared. Three main clusters were defined in the studied group of strains (Figure 3). The Italian and Egyptian strains were located with the strain from Tunisia in the first cluster (Cluster I). All the Greek strains and some of the Portuguese strains with a strain from Italy belonged to the second cluster (Cluster II), and the rest of the Portuguese strains and one Italian strain were from a third cluster (Cluster III) (Figure S1). An Egyptian *B. melitensis* strain isolated from cattle in 2011 (Egypt 29) was allocated in the same sub-cluster with a strain isolated from a bovine in Italy (Italy 33) in the same year and with an Italian strain (Italy 43) isolated from a man in 2013. The Egyptian strain showed low genetic diversity in two loci (*Bruce*06: 2→3 and *Bruce*16: 6&7→4). A *B. melitensis* strain isolated from sheep in 2014 in Egypt (Egypt 32) showed a similar genotype as an Italian strain (Italy 39) isolated from sheep in 2011 (only differing in *Bruce*07: 6→5, and *Bruce*19: 42→43). The only *B. melitensis* strain from Tunisia was allocated in the same sub-cluster with the Egyptian and Italian strains. Two closely related human strains were found, one isolated from Italy in 2011 (Italy 38) and one isolated from Greece (Greece 44). Both are allocated in the same sub-cluster with low genetic diversity represented by one difference in the number of repeats at loci *Bruce04, Bruce*16, and *Bruce*30. One Italian *B. melitensis* strain (Italy 40) isolated from a goat in 2011 was similar to a strain isolated from a sheep in 2006 in Portugal (Portugal 38), and low genetic diversity was represented by one difference in the number of repeats in one locus (*Bruce*09: 6→7). A *B. melitensis* isolated from a man in Greece (Greece 19) was similar to a strain recovered from a sheep in Portugal (Portugal 36) in 2005, showing a low genetic diversity in loci *Bruce*16 and *Bruce*07. The described relations between the 163 Mediterranean strains are shown in Supplementary Materials Figure S3.

**Figure 4:**
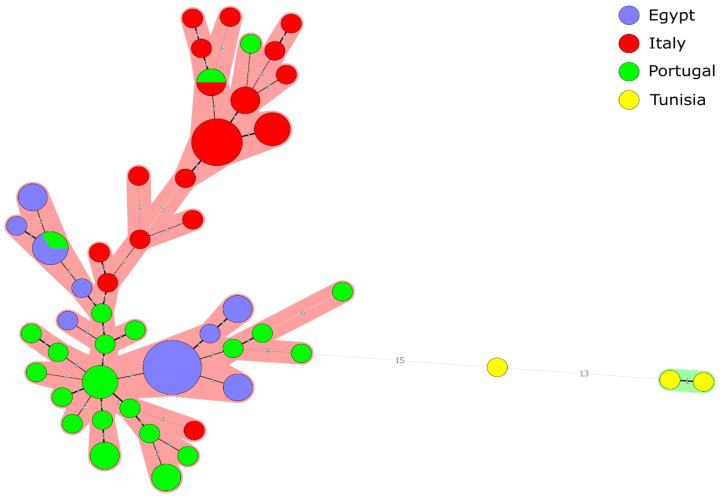
MLVA-16 minimum spanning tree describing the relationships of 74 *B. abortus* isolates. Circles represent MLVA-16 genotypes, colored according to the country of origin, and the size of the circle indicates the number of strains with that genotype.

The results of the MLVA-16 profiles of 74 *B. abortus* strains from Egypt (*n* = 20), Italy (*n* = 25), Portugal (*n* = 26), and three strains from Tunisia have been investigated and compared. No *B. abortus* strains were available from Greece. Two main clusters have been defined in the studied group of strains (Figure 4). All the Egyptian and Tunisian strains, most of the Portuguese strains and some of the Italian strains were located in the first cluster (Cluster I). Most of the Italian strains and two of Portuguese strains formed the second cluster (Cluster II). In general, Egyptian *B. abortus* strains isolated from cattle are similar to or located in the same sub-cluster with strains isolated from cattle in Portugal and Italy. For instance, two *B. abortus* strains isolated from cattle in 2012 and 2014 (Egypt 10, 12) in Egypt have the same genotype of a *B. abortus* strain isolated from cattle in Portugal (Portugal 25) in 2005. In addition, an Egyptian *B. abortus* strain (Egypt 11) recovered from cattle in 2011 was similar to a strain recovered from cattle from Portugal in 2001 (Portugal 15) with very low genetic diversity represented by one difference in the number of repeats at one locus (*Bruce*43: 2→3). Moreover, a strain recovered from cattle in 2004 in Portugal (Portugal 5) presented the same genotype of a strain recovered from cattle in Italy in 2011 (Italy 11). A strain recovered from cattle in Portugal in 2007 (Portugal 4) was allocated in the same sub-cluster and similar to a strain recovered from cattle in Italy in 2011 (Italy 1) with low genetic diversity in two alleles (*Bruce*07: 6→7 *Bruce*09: 7→5). One strain recovered from cattle in Portugal in 2007 (Portugal 22) was similar to a strain recovered from buffalo in Italy in 2011 (Italy 6) with one difference in one allele (*Bruce*16: 3→5). The described relations among the 74 Mediterranean strains are shown in the Supplementary Materials Figure S4.

## 3. Discussion

**Figure 5:**
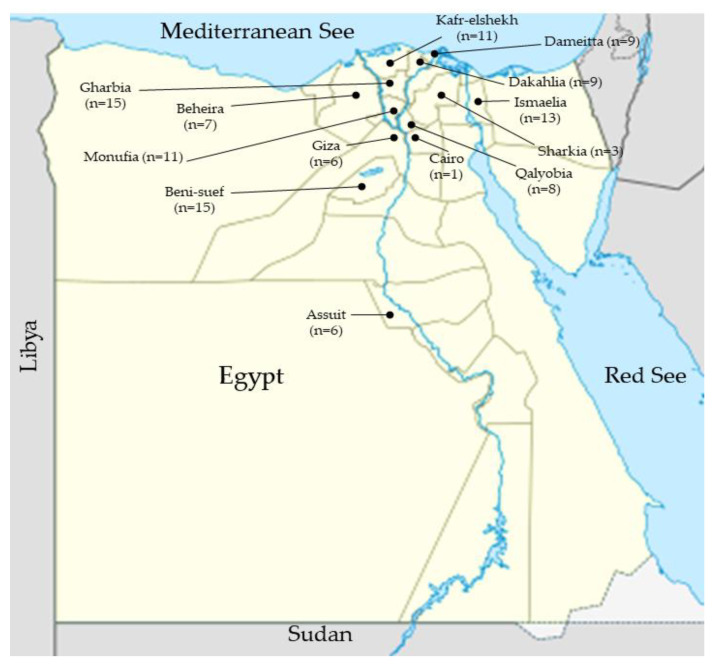
Map of Egypt showing the specific governorates and the number of strains collected from each governorate.

Brucellosis is a zoonosis of worldwide distribution. The disease is endemic in the Mediterranean basin, but accurate epidemiological data are not available for most of the area’s countries [12]. In Egypt, the disease is notorious in livestock, but it is a neglected disease in human medicine. The national surveillance program of the General Organization of Veterinary Service (GOVS) gave indirect proof of brucellosis in bovines, small ruminants, and camels in 22 of 27 administrative Egyptian governorates using Rose Bengal test (RBT) and slow agglutination test (SAT) tests [18]. As expected, *B. melitensis* and *B. abortus* strains could be isolated from all farm animal species in 13 administrative governorates representing the main geographical regions of Egypt (Figure 5). The ubiquitous occurrence of *B. melitensis* in bovines confirms its ability to cross species barriers and to establish permanent reservoirs in cattle and buffaloes as previously found for Albania, France, Israel, Italy, and Turkey [12]. This can also be assumed for Egypt. Cross-species transmission of the small ruminant pathogen *B. melitensis* to bovines, camels, and Nile catfish was reported to occur in Egypt [8,20,21]. The majority of cattle and buffaloes in Egypt are owned by individual households, are kept in small, mixed herds, and are moved daily between the house yard and grazing lands. Circulation of *B. melitensis,* the most virulent species for humans, in cattle and buffaloes increases the risk for human infection. Isolation of *B. abortus* from dogs and cats has highlighted the biological role of carrier hosts in the re-emergence and dissemination of the infection [10]. This complicated epidemiological situation will result in difficulties for surveillance and control programs of brucellosis in Egypt.

The genus *Brucella* consists of highly homogenous and highly monomorphic species of bacteria with minimal genetic variation. The classification of *Brucella* relies mainly on differences in pathogenicity and host specificity in combination with classical identification based on biochemical characteristics, i.e., CO_2_ requirement, H_2_S production, urea hydrolysis, dye sensitivity or immunological characteristics as agglutination with monospecific anti-sera, and phage lysis. These criteria are unable to trace back the origin of *Brucella* or to discriminate among strains effectively [22]. Thus, MLVA was applied to evaluate the epidemiological relationships of *B. melitensis* and *B. abortus* isolates recovered from different livestock species in different Egyptian governorates. The MLVA-16 is a tool of choice to profile highly homogenous populations of *Brucella* in a country, and it was successfully used as a powerful tool to discriminate *Brucella* isolates even on a global scale [23]. The different genotypes of brucellae are heterogeneously distributed over time and space in Egypt. This finding suggests that brucellae maybe endemic for a long time in Egypt and spread slowly across the nation with trade of animals or products or have been introduced in waves with large numbers of animals that were distributed nationwide in short periods. Both scenarios are likely for the Egyptian setting. In the remains of a diseased human in a pharaonic tomb, brucellae were found, proving the circulation of such strains for thousands of years. On the other hand, every year, the uncontrolled movement of animals among different governorates takes place. Particularly, during “Eid al-Adha”, the biggest Islamic fest, thousands of animals are moved among different administrative governorates for slaughtering which might not contribute to the spread directly. Open, mixed animal markets nationwide without veterinary inspection result in the spread of various diseases including brucellosis.

According to the MLVA-16 profiles, all tested *B. melitensis* strains showed complete homogeneity in the panel-1 markers that are used for species assignment of strains, and no differences were found for strains collected from different animal species as well as from different governorates. Similar results obtained in the MLVA-16 profiles of *B. abortus* with a minor diversity in loci *Bruce*06 and *Bruce*43. Thus, a typical Egyptian cluster exists. Loss of two repeat units in *Bruce*06 might be a regularly occurring event and already happened in our laboratory, at least in *B. abortus* RB51. Our observation corroborated with the findings of Dorneles et al. [24], who observed the addition and deletion of one repeat unit in *Bruce*07 of *B. abortus* S19 and RB51 vaccine batches after ten serial passages [24].

Genotyping analysis of *B. melitensis* isolates collected from human cases in Egypt has been done previously with MLVA-15 [25]. The isolates were collected from acute-febrile illness cases from 1999 to 2003. On the other hand, brucellae from livestock were investigated using MLVA-15, but this investigation was done only for four limited geographical areas with a limited number of isolates (*n* = 17) including only two *B. abortus* strains [26]. However, MLVA-15 indicating a high genetic diversity among the tested 13 *B. melitensis* strains with eight different genotypes. Recently, the genetic variation of twelve *B. melitensis* and six *B. abortus* strains isolated from Egypt between 2002–2013 have been investigated by MLVA-16. The strains revealed eleven and three genotypes, respectively. *Brucella abortus* isolates were closely related to Western, Mediterranean, and East Asian strains and clonal lineages from the Americas, and *B. melitensis* isolates were mostly closer to Western and Eastern Mediterranean clonal lineages [27]. A comprehensive genotyping of 118 Egyptian *B. melitensis* bv3 utilizing MLVA-16 showed correlation to the West Mediterranean lineage. The strains showed high diversity discriminated into 70 genotypes; of them, 51 genotypes were represented by single isolates [28]. To the best of our knowledge, this study is the first MLVA-16 analysis with a significant number of brucellae (*B. abortus* and *B. melitensis*) from cattle, buffaloes, sheep, goats, camels, dogs, and cats isolated from 13 endemic administrative governorates in Egypt. It is also the first MLVA-16 analysis providing data on *B. melitensis* strains from Greece. Contagious zoonotic diseases, such as brucellosis, cross national and international boundaries with ease, and MLVA-16 can help to trace these movements forwards and backwards efficiently. Indeed, Egyptian *B. melitensis* strains characterized in the present work showed an MLVA pattern closer to the Italian strains with very low genetic diversity, and Italian strains have a similar genotype to isolates collected from Portugal and Greece. A part of the Portuguese strains belongs in the same cluster with the Greek strains although the two countries are geographically separated (West and East Europe). The Egyptian strains belong to the same cluster with Italian strains and Tunisian. This may be due to the movement of animals and animal products in the trades or occupations that occurred over the past century. On the other hand, *B. abortus* strains that originated in Portugal were found to be similar or had the same genotype as strains originating from Egypt and Italy. These results are, to some extent, in agreement with the data obtained by Lounes et al. [29], who demonstrated that there is a lineage between *Brucella* strain isolated from the Maghreb and European strains due to the long-lasting socio-historical connections of Africa with Europe [29]. The results in the present study are consistent also with previous observations of Kay et al. [16], who demonstrated that the persistence of *Brucella* infection and specific lineages in the Mediterranean region over time might be possible [16].

The majority of *B. melitensis* strains was assigned to biovar 3, which has been reported to be the most predominant biotype isolated from humans and animals in the Mediterranean region [12]. We additionally observed that strains belonging to two different biovars were closely related, having the same MLVA profile. Classical typing methods are fatigued and require skilled personnel and are prone to errors more than molecular typing techniques. Thus, incorrect biovar detection can happen. Serology and PCR-based assays represent applicable and very efficient methods for the diagnosis of brucellosis [30]. However, it can be assumed that the results of those traditional biotyping tools may not strictly reflect the genetic or phylogeographic clustering of *B. melitensis.* Forty isolates were not included in the analysis because of missing VNTR calls. Two loci, *Bruce*19 and *Bruce*07 were missing in all excluded strains. The PCR product of the two loci were not seen during the experiment. Missing in VNTR could be due to the negative amplification that may result from the lack of a VNTR locus in the isolates or because of technical failure. Negative amplification of loci has previously been reported during MLVA16 typing of *B. melitensis* and *B. abortus* isolates [31,32]. The MLVA based on PCR is laborious and requires trained personnel. The recently implemented whole genome sequencing (WGS) typing methods provide higher resolution genetic clustering and can overcome the drawbacks of missing VNTR calls. Thus, higher resolution molecular tools based on next-generation sequencing (NGS) technology are now to be preferred and required for epidemiological studies and identification of the outbreaks of *Brucella* [33]. The application of core-genome multilocus sequence typing (cgMLST) and SNP analysis provided a higher phylogenetic distance resolution than MLVA for *B. melitensis* isolates belonging to the same lineage. This helped in the accurate typing, identification, clustering, and distinguishing of diverse and unrelated genotypes [34,35].

## 4. Materials and Methods

### 4.1. Brucella Strains

One-hundred and nine *Brucella* isolates were cultured from different animal species in Egypt randomly. The isolates were collected between 2011 and 2017. The strains were cultured from seropositive cattle (*n* = 52), buffaloes (*n* = 29), sheep (*n* = 14), goats (*n* = 9), and one isolate from a camel, a dog, and a cat each. Two strains of *B. abortus* RB51 vaccine were cultured from vaccine vials (CZ Veterinaria, Spain) used for animal immunization in the Delta region of the Nile. All field strains were associated with a history of abortion or reproductive failure. The isolates were obtained from stomach contents and spleens of the aborted fetuses, from lymph nodes, milk, vaginal discharge, and uterine excreta of the aborted cows and dams. As shown in Table 2, the isolates were collected from 13 governorates representing the metropolitan capital cities, Cairo and Giza (*n* = 7), the North coastal governorates (*n* = 18), the Northeastern part of the country (*n* = 13), Upper Egyptian governorates (*n* = 21), and the Delta region governorates (*n* = 50) (Table 2). Each strain in the current study was isolated and represented only one animal but maybe from the same herd. After preliminary identification of strains at the genus level, all isolated brucellae were sent to the OIE reference laboratory for brucellosis at Friedrich–Loeffler–Institut, Jena, Germany for biotyping and genotyping.

### 4.2. Identification and Biotyping of Isolates

The *Brucella* species identification was carried out using matrix-assisted laser desorption/ionization (MALDI-TOF-MS) as previously described [36]. In brief, single colonies were collected from the pure culture in 300 µL of HPLC grade water and then vortexed and inactivated using 900 µL of absolute ethanol. The whole protein contents extracted by formic acid and acetonitrile and then spotted on MALDI plate and overlaid with saturated α-cyano-4-hydroxycinnamic acid matrix solution (in 50% acetonitrile and 0.25% trifluoroacetic acid) as described before [37]. The MALDI measurements were carried out using a Microflex LT (Bruker Daltonics, Bremen, Germany). The MALDI Biotyper manufacturer’s recommendation on the log score value of 0–3 for species identification was followed. Score values more than 2.3 indicate “highly probable species identification”; values between 2.0 and 2.29 indicate “probable species identification”; values between 1.7 and 1.99 indicate “probable genus identification”; and values less than 1.69 indicate “no reliable identification”. The species identification was accepted if the score of 2.3 or higher was obtained.

Biotyping of *Brucella* isolates carried out according to colony morphology, biochemical reactions (oxidase, catalase, urease), CO_2_ requirement, production of H_2_S, growth in the presence of thionin and fuchsine dyes, reaction with mono-specific anti-sera (A, M, R), agglutination with trypaflavine and crystal-violet, and phage lysis (wb, Tb, F25) as described by Alton [38]. Genomic DNA extracted from heat-inactivated biomasses by using the High-Pure template preparation kit (Roche Applied Sciences, Mannheim, Germany) according to the manufacturer’s instructions. DNA content of samples was measured, and *Brucella* species were molecularly confirmed by AMOS-PCR [39].

### 4.3. Genetic Diversity of Brucella Strains Analyzed MLVA-16

Genotyping of *Brucella* strains was performed using MLVA-16 according to Le Flèche et al. (2006) [40] with the modifications made by Al Dahouk et al. (2007) [22]. The assay comprises two panels; panel-1 contains eight moderately variable minisatellite markers (*Bruc*e06, *Bruc*e08, *Bruc*e11, *Bruc*e12, *Bruc*e42, *Bruce*43, *Bruc*e45, and *Bruce*55), used to trace back the geographic origin of strains; and panel-2, constituted by eight highly polymorphic microsatellite markers, useful for outbreak investigation. Panel-2 was further divided into panel-2A with three loci (*Bruc*e18, *Bruc*e19, and *Bruc*e21), and panel-2B with five loci (*Bruc*e04, *Bruc*e07, *Bruc*e09, *Bruce*16, and *Bruc*e30). The number of repeats at each locus was determined by the correlation with the amplicon size according to the 2013 *Brucella* allele assignment table (Le Flèche et al. (2006) [40], version 3.6 available at MLVA bank for microbes genotyping (http://mlva.u-psud.fr). Genomic DNA from *B. melitensis* bv1 strain 16M was used as a control for alleles assignment. Cluster analysis of MLVA-16 data was based on the categorical coefficient and unweighted pair group method with arithmetic averages (UPGMA) using the BioNumerics software package (Applied Maths, Belgium). Neighbor-joining cluster analysis for the MLVA-16 profiles of the *B. melitensis* and *B. abortus* isolates was performed. The genetic diversity for Egyptian *B. melitensis* and *B. abortus* strains were calculated, and the results were compared with those isolated from different animal species and humans from different Mediterranean countries. Only 69 Egyptian strains (49 *B. melitensis* and 20 *B. abortus*) were used in the comparison. While the other 40 strains were excluded from the comparison because the number of repeats cannot be determined and missed in at least two loci in each strain (Supplementary Materials Table S1). In total, 237 Mediterranean strains obtained from Egypt, INIAV in Portugal, VRI of HAO-DEMETER in Greece, IZS in Italy and IRVT in Tunisia were used for comparison. All strains used in the current study belonged to the authors’ culture collection, neither published nor deposited in a public database, except some of the Portuguese *B. melitensis* and *B. abortus* strains presented in this work were used in a preview work [31]; however, for consistency, all procedures of the work were carried out again for all strains. Identification of either the Egyptian or the other Mediterranean strains were carried out by MALDI-TOF [36], standard bacteriology, and biochemical tests as previously described in Section 4.2. according to Alton [38]. The DNA extraction and confirmation of isolates at the species level was done utilizing AMOS-PCR as previously described [39]. We carried out MLVA-16 for all strains at our laboratory as mentioned above. The MLVA-16 genotypes of the 69 isolates from Egypt was compared to those from Italy (*n* = 49), Greece (*n* = 63), Portugal (*n* = 52), and four strains from Tunisia (Table 3).

## 5. Conclusions

Brucellosis is a notorious disease in veterinary medicine, while it is often neglected in human health. *Brucella melitensis* is the predominant species circulating in livestock, has successfully crossed host–species barriers, and has established new reservoirs in non-specific hosts. Several factors have contributed to this cross-species transmission and transnational dissemination. Insufficient implementation of control systems leading to the mixing of infected animals at the village level and during grazing may have led to the spread of infection to healthy herds in Egypt. Open, mixed animal markets must be controlled efficiently by veterinary public health. Brucellae from the Mediterranean basin have close genetic relatedness, independent of the country of origin, but show astonishing coherence of MLVA 16 clusters. The classical molecular tools and conventional typing techniques for brucellae are not always satisfactory for subtyping and epidemiological tracing. However, MLVA-16 is a powerful tool to rapidly assess the genetic diversity of bacterial populations on a large scale allowing intercontinental cross-border tracing. It will be amended by whole-genome sequencing in the near future.

## Figures and Tables

**Table 1:** Numbers and classification of *Brucella* spp. isolates from different animal hosts in Egypt collected from 2011–2017.

Host	Cattle	Buffalo	Sheep	Goat	Camel	Dog	Cat	Vaccine	Total
*B. melitensis*	34	27	14	9	1	0	0	0	85
*B. abortus*	18	2	0	0	0	1	1	2	24
Number of strains	52	29	14	9	1	1	1	2	109

**Table 2:** Numbers and geographical distribution of *Brucella* strains recovered from animals in Egypt collected from 2011–2017 (A: *B*. *abortus*; M: *B*. *melitensis*).

No.	Geographical Area	Governorates	Number of Isolates
1	Capital cities governorates	Giza	6 (1A, 5M)
		Cairo	1 (1M)
2	The North coastal governorates	Dakahlia	9 (8A, 1M)
		Dameitta	9 (5A, 4M)
3	The Northeastern part of the country	Ismaelia	13 (1A, 12M)
4	Upper Egypt governorates	Assuit	6 (6M)
		Beni-suef	15 (3A, 12M)
5	Delta region governorates	Beheira	7 (6A, 1M)
		Gharbia	15 (15M)
		Monufia	11 (11M)
		Qalyobia	8 (8M)
		Sharkia	3 (3M)
		Kafr-elshekh	6 (6M)
Total number		13	109 (24A, 85M)

**Table 3:** Numbers, classification, details information, and the countries of origin of the *Brucella* spp. isolates used for comparison.

Country	*Brucella* spp.	Host	Years of Isolation	No. of Isolates
Egypt (*n* = 69)	*B. abortus*	Cattle, buffalo, dog, cat, RB51 vaccine strain	2011–2017	20
	*B. melitensis*	Cattle, buffalo, sheep, goat, camel	2011–2017	49
Italy (*n* = 49)	*B. abortus*	Cattle, buffalo	2011–2015	25
	*B. melitensis*	Sheep, goat, bovine, humans, ibex	2011–2016	24
Portugal (*n* = 52)	*B. abortus*	Cattle	2001–2007	26
	*B. melitensis*	Sheep, goat, cattle, human	2001–2010	26
Greece (*n* = 63)	*B. abortus*	-	-	0
	*B. melitensis*	Human, small ruminants	ND *	63
Tunisia (*n* = 4)	*B. abortus*	Cattle	2018	3
	*B. melitensis*	Sheep	2017	1
Total			2001–2017	237

* ND: not determined.

## Supplementary Materials

The following are available online at https://www.mdpi.com/2076-0817/9/6/498/s1. **Figure S1:** The similarity matrix showing the similarity percentages and the maximum and minimum number of identical or different VNTRs within the cluster and between the two distinguished clusters of 49 *B. melitensis* isolates recovered from animal in Egypt. **Figure S2.** The similarity matrix showing the similarity percentages and the maximum and minimum number of identical or different VNTRs within the cluster and among three distinguished clusters of 20 *B. abortus* isolates recovered from animal species in Egypt. **Figure S3:** Dendrogram based on MLVA-16 genotyping (UPGMA method) showing the relationship between 163 *B. melitensis* isolates originated from the Mediterranean basin. **Figure S4:** Dendrogram based on MLVA-16 genotyping assay (UPGMA method) showing the relationship between 74 *B. abortus* isolates originated from the Mediterranean basin. **Table S1:** MLVA-16 profile and the metadata of the 40 excluded *Brucella* strains due to loss number of repeats in at least two loci in each species. The non-determined number of repeats is referred to as (ND) in the table.
