# Supplementary figures and images for: MLVA-16 Genotyping of Brucella abortus and Brucella melitensis Isolates from Different Animal Species in Egypt: Geographical Relatedness and the Mediterranean Lineage
Source: Pathogens. 2020 Jun 22;9(6):498. doi: 10.3390/pathogens9060498 (PMC7350383; doi:10.3390/pathogens9060498)

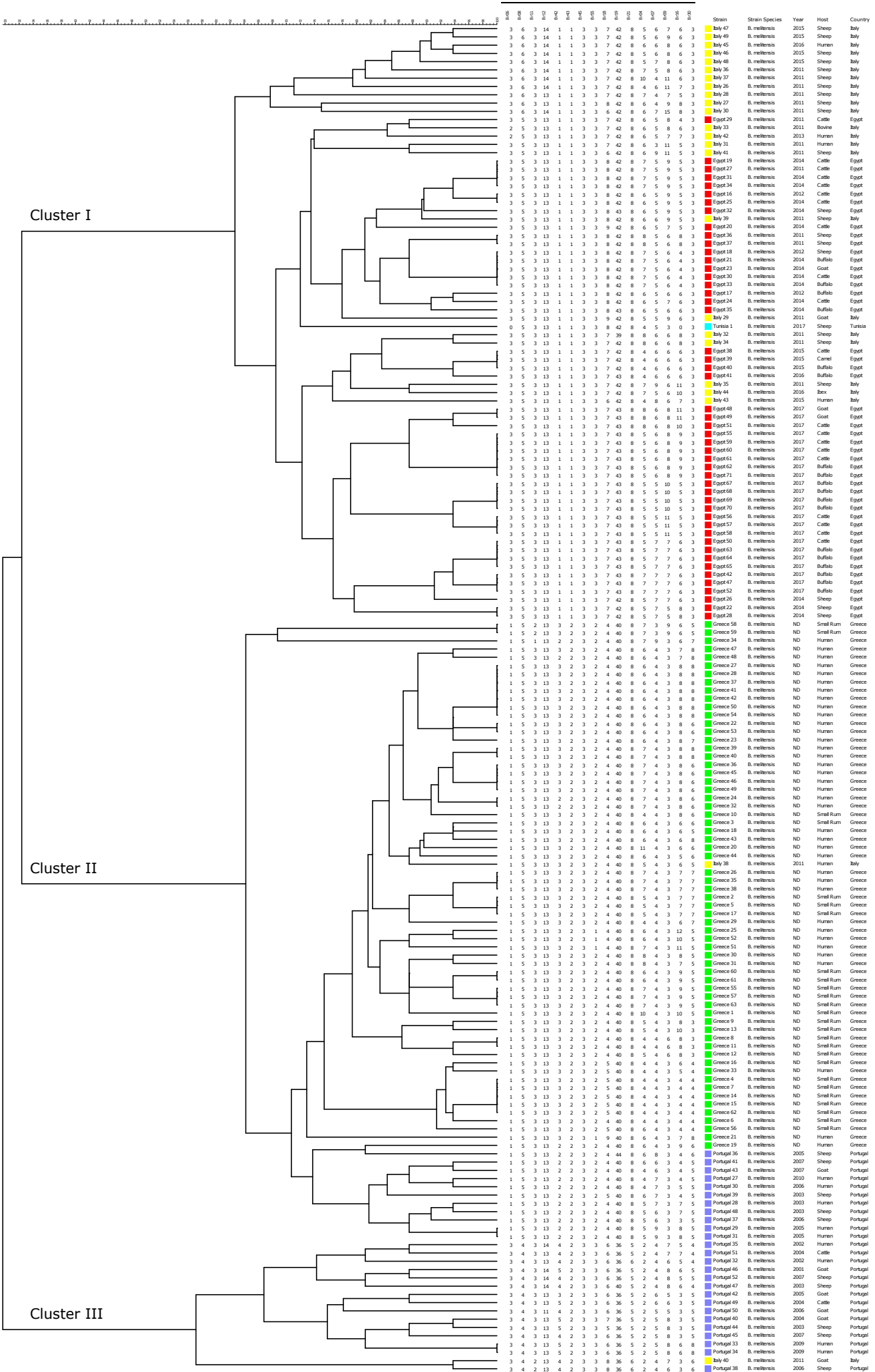

Supplement: Supplementary file 1 [file pathogens-09-00498-s001.zip › Figure S3.pdf]

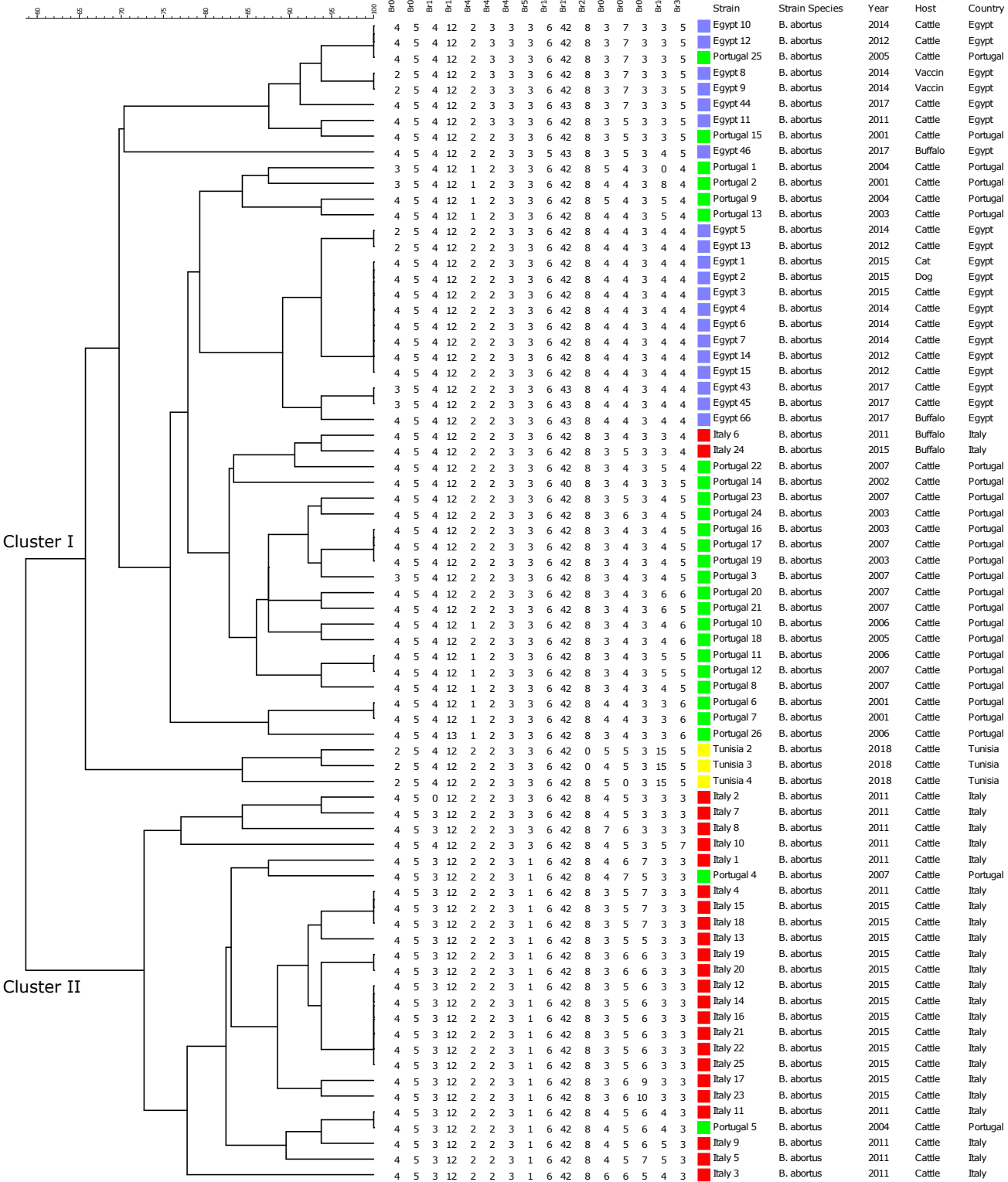

Supplement: Supplementary file 1 [file pathogens-09-00498-s001.zip › Figure S4.pdf]
